# High VISTA expression is linked to a potent epithelial-mesenchymal transition and is positively correlated with PD1 in breast cancer

**DOI:** 10.3389/fonc.2023.1154631

**Published:** 2023-04-20

**Authors:** Ibtissam Rezouki, Basma Zohair, Saadia Ait Ssi, Mehdi Karkouri, Ibtissam Razzouki, Mohamed Elkarroumi, Abdallah Badou

**Affiliations:** ^1^ Laboratory of Immunogenetics and Human Pathologies, Faculty of Medicine and Pharmacy of Casablanca, Hassan II University, Casablanca, Morocco; ^2^ Laboratory of Pathological Anatomy, University Hospital Center (CHU) Ibn Rochd, Hassan II University, Casablanca, Morocco; ^3^ Department of Obstetrics and Gynecology, University Hospital Center (CHU) Ibn Rochd, Casablanca, Morocco; ^4^ Mohammed VI Center for Research and Innovation, Rabat, Morocco, and Mohammed VI University of Sciences and Health, Casablanca, Morocco

**Keywords:** VISTA, breast cancer, immune checkpoint, epithelial-mesenchymal transition, PD1, immunosuppressive microenvironment

## Abstract

Breast cancer is the most common type of tumor in women worldwide. Immune checkpoint inhibitors, particularly anti-PDL1, have shown promise as a therapeutic approach for managing this disease. However, this type of immunotherapy still fails to work for some patients, leading researchers to explore alternative immune checkpoint targets. The Ig suppressor of T cell activation domain V (VISTA) has emerged as a novel immune checkpoint that delivers inhibitory signals to T cells and has demonstrated encouraging results in various cancers. Our study investigated the association of VISTA expression with clinicopathological parameters in breast cancer patients, its involvement in the Epithelial-Mesenchymal-Transition (EMT) process, and its correlation with PD1 expression. Transcriptomic analysis revealed that VISTA was associated with lobular and metaplastic histological type, tumor size, lymph node status, ER and PR negative status, and the TNBC molecular subtype. Furthermore, VISTA expression was strongly associated with an immunosuppressive tumor microenvironment. Immunohistochemistry analysis corroborated the transcriptomic results, indicating that VISTA was expressed in most immune cells (94%) and was significantly expressed in breast cancer tumor cells compared to matched adjacent tissues. Our study also showed for the first time that VISTA overexpression in breast cancer cells could be associated with the EMT process. Additionally, we identified a positive correlation between VISTA and PD-1 expression. Together, these results highlight the immunosuppressive effect of VISTA in breast cancer patients and suggest that bi-specific targeting of VISTA and PD-1 in combination therapy could be beneficial for these patients.

## Introduction

Despite therapeutic advances, breast cancer (BC) remains the most prevalent cancer, accounting for 11.7% of all new cancer cases (1). Considering the heterogeneous aspects of breast tumors, the therapeutic approach relies chiefly on the administration of endocrine therapy for hormone-sensitive cancers, anti-HER2 targeted therapy for HER2-enriched tumors, and chemotherapy, which constitutes the gold standard treatment for TNBC. Unfortunately, despite all currently available treatments, clinical benefit and efficacy remain quite limited in a large number of patients, and up to 30% of recurrences and breast metastases involve therapeutic resistance (2–6).

It has been reported that epithelial-mesenchymal transition (EMT) might lead to treatment resistance in breast cancer patients (7–9). Given its association with adverse clinical prognostic features such as estrogen receptor (ER) negative status and tumor progression, the EMT process reveals its detrimental impact on patient prognosis (10). These findings were supported by studies demonstrating the upregulation of mesenchymal markers and downregulation of epithelial markers within aggressive breast cancer subtypes, notably triple-negative or basal-like cancer (11). EMT is mainly maintained by tumor tissue inflammation, impeding consequently the antitumor immune response through inhibitory immune checkpoint activation (9, 12). Although blocking these molecules, mainly CTLA-4, PD-1 and PD-L1, has emerged as a promising immunotherapeutic approach, patients with the most aggressive breast tumor subtypes still exhibit poor survival (13–15). It is therefore necessary to explore alternative immune checkpoint molecules to improve breast cancer patient survival.

In recent years, there has been increasing interest in VISTA as a potential new target for several types of cancer. The notable feature of this immune checkpoint is its expression in both activated and naïve T cells (16). Moreover, VISTA upregulation has been linked to increased regulatory T cells and worse survival rates (17, 18). Several studies have highlighted VISTA upregulation following PD-1/L1 or CTLA-4 blockade, suggesting that VISTA might contribute to immune checkpoint inhibitor resistance (19, 20). In light of these observations, inhibiting the VISTA pathway may be the key to improving antitumor immune responses and preventing resistance to current immunotherapies. These facts prompted us to investigate VISTA expression in the tumor microenvironment of breast cancer patients.

T cell activation domain V-containing Ig suppressor (VISTA), also known as PD-1 homolog (PD-1H), differentiation of embryonic stem cells 1 (Dies1), C10orf54, platelet receptor Gi24 precursor, DD1, SISP1 and B7-H5 (21–23), is highly expressed on various immune cells, including NK cells, naive CD4+ T cells, CD8+ T cells, regulatory T cells (Tregs), as well as on CD11b high myeloid cells, including granulocytes, monocytes, macrophages and myeloid dendritic cells (DCs) (24–26). This molecule may have a dual role in inhibiting T cell activation notably as a ligand or as a receptor (27–29). At normal pH, VISTA acts as a receptor by interacting with its ligand, V-set and immunoglobulin domain containing 3 (VSIG-3), also known as IGSF11 (29, 30). In contrast, at acidic pH, VISTA functions as a ligand expressed on tumor cells and binds t receptor known as P-selectin glycoprotein ligand-1 (PSGL-1) (28). Multiple studies have given an overview of the key role of VISTA in immune regulation. A growing body of research has revealed that the pharmacological blockade of VISTA triggers the reactivation of the immune response by increasing the production of antitumor cytokines and chemokines such as IFN, IL-2, IL-17, CCL-5, CCL-3 and CXCL11 (29, 31). Moreover, several types of cancer have presented an upregulation of VISTA within the tumor microenvironment (32). Of note, this protein appears to have immune-inhibitory properties in various cancers, including melanoma, prostate cancer, renal cell carcinoma, non-small cell lung cancer, acute myeloid leukemia, colorectal cancer, brain cancer, ovarian and endometrial cancers (19, 20, 33–40),. Consequently, VISTA overexpression results in worse overall survival (OS) with suppression of TCD8+ cell proliferation, a decrease in antitumor cytokine production, as well as significant Treg infiltration.

In breast cancer, the role of VISTA expression has produced conflicting results. The first study to examine VISTA expression in the mammary tumor microenvironment revealed that immune cells expressing high levels of this protein positively correlate with prolonged relapse-free survival (RFS) and overall survival (OS), predicting a better prognosis for breast cancer patients (41). Conversely, another study reported that VISTA is highly expressed on tumor-associated macrophages (TAMs) and is associated with poor clinical outcomes (42, 43).

On the other hand, combination therapy targeting multiple immune checkpoints appears to be an effective strategy for improving cancer immunotherapy. It has been shown that simultaneous blockade of VISTA and PD-1 has synergistic effects on restoring T cell effector functions (44, 45).

In light of these insights, our study aims to explore the expression profile of VISTA in breast cancer patients, its association with EMT markers and its correlation with PD-1. Our results suggest that VISTA may be a promising immunotherapeutic target in breast cancer, particularly in patients resistant to FDA-approved immune checkpoint inhibitors.

## Materials and methods

### Patients and samples

A total of 55 Biopsy samples, including tumor tissues and uninvaded adjacent tissues, were obtained from breast cancer patients who underwent surgery at University Hospital Center Ibn Rochd of Casablanca. Clinicopathological data, such as age, grade, histological type, molecular subtype, TNM staging and overall survival, were collected for each patient. The regional ethics committee approved the use of patient samples and corresponding data for this research. Additionally, informed consent was obtained from patients recruited for the study.

### TCGA and TISCH data analysis

We assessed RNA-seq data and clinicopathological parameters of 1,084 patients diagnosed with invasive breast carcinoma from The Cancer Genome Atlas (TCGA). The data were downloaded from open-access databases (https://www.cbioportal.org/). A pretreatment phase of the transcriptomic dataset was performed, followed by the grading of patients according to the WHO classification, and conversion of all gene expression patterns to log2 format.

For a complete exploration of immune checkpoint expression (VISTA, PD1, PDL1, and CTLA4) on the immune and stromal cells in breast cancer, we used the Tumor Immune Single Cell Hub (TISCH)-based GSE114727_inDrop data. The data were downloaded from an open-access database (http://tisch.comp-genomics.org/).

### Extraction and qRT-PCR

Total RNA was extracted from fresh biopsies, including tumor and adjacent uninvaded tissues, of 55 breast cancer patients and stored in TRIsure (Bioline GmbH, Germany) at -80 C. After adding 1mL of TRIsure reagent, the biopsy was ground, and 160 μL of chloroform was added (Carlo Erba Reagents, France). The aqueous phase was transferred to a fresh tube containing 400 μL of isopropanol (PanReac AppliChem, Germany) and centrifuged at 12,000 g. The resulting pellet was suspended in ultrapure water after being washed with 800 μL of 75% ethanol (Biosmart, Morocco). RNA quality and quantity were assessed using a NanoVueTM Plus Spectrophotometer (GE Healthcare, UK).

We synthesized cDNA from 1 μg of total RNA using the following reaction mixture: 1 µL Random Hexamer Primer 25 µg (Bioline, France), 0.5 µL of RNase inhibitor (Invitrogen, France), 0.5 µL of Tetro Reverse Transcriptase enzyme (Bioline, France), 4 µL of Tetro Reverse Transcriptase buffer,4 µL of dNTP (10 mM).

The volume was completed with ultrapure water. The reaction mixture was then placed in a thermocycler (Society, Country), and reverse transcription was performed according to the following program: 25°C for 10 min 42°C for 45 min, and 70°C for 15 min, followed by 4°C.

SYBER Green was used in real-time PCR to measure the relative expression of the VISTA gene (10 μM) compared to the expression of the internal control gene β-actin at a concentration of 10 μM.

**Table:** 

Genes	Forward Sequences	Reverse Sequences
β-actin	5′-TGGAATCCTGTGGCATCCATGAAAC-3′	5′-TAAAACGCAGCTCAGTAACAGTCCG-3′
VISTA	5-TGTAGACCAGGAGCAGGATG-3′	5-ATGCACCATCCAACTGTGTG-3′

The Bio-Rad Real-Time PCR System was used to conduct the PCR. Initially, we prepared the mixture consisting of 10 µL of SYBR Green, 7 µL of H2O, and 0.5 µL of each primer sequence (Forward and Reverse). After adding 2 µL of cDNA to each well of the PCR plate, and 2 µL of ultra-pure water for the negative control, we added 18 µL of the mixture and performed the PCR according to the established program.

The PCR was programmed as follows: a 10-minute incubation at 95°C was used to activate the polymerase and denature the material, followed by 40 cycles of 15 seconds at 95°C for annealing and 60 seconds at 60°C for an extension. Cycle threshold (Ct) values were estimated using fluorescence data collected after each cycle’s extension phase. Finally, to determine the relative expression of the genes in each sample, we used the Ct values to calculate the function (2−ΔΔCt).

### Immunohistochemistry (IHC)

A total of 51 breast tumor samples with different molecular subtypes (Luminal B, Luminal A, HER2+, and TNBC) and 8 adjacent uninvolved tissue samples were preserved in formalin solution and embedded in paraffin. The tissues were sectioned to obtain 3-4 μm thick slices for immunohistochemistry analysis (IHC). The sections were oven-dried for one hour at 60°C, and then overnight at 37°C before processing. The antigenic sites were then deparaffinized, rehydrated, and unmasked using the PT link device, which performs a 3-in-1 pre-treatment using EnVision FLEX Target Retrieval Solution, High pH (50X). The sections were washed three times and treated with EnVision FLEX Peroxidase-Blocking Reagent (Dako, Denmark) to inhibit endogenous peroxidase. They were then incubated with 10 μg/ml of VISTA (C10orf54) Mouse Monoclonal Antibody [Clone ID: UMAB271] (OriGene Technologies, Inc, USA), and PD-1 (DBM15.5) Mouse Monoclonal Antibody [Clone ID: Mob475] (Diagnostic BioSystems Inc, USA) at 1:100 dilution, along with their respective isotype control IGg1 (Mouse IgG1 Isotype Control Antibody | Clone ID: MOPC-21-LS-C355904) at 5μg/ml (LifeSpan BioSciences, Seattle, United States) (40). EnVision FLEX/HRP solution (Dako, Denmark) was added, and the slides were incubated in the dark for 20 minutes. For the revelation step, incubation was performed for 10 minutes following the addition of the DAB Substrate-Chromogen working solution (Dako, Denmark). Finally, the slides were kept in Hematoxylin for 3 to 5 minutes before being cleaned in three toluene baths for 1 minute each.

### Evaluation of immunostaining

The intensity of labeling on tumor cells (TC) and immune cells (IC) was independently evaluated by two pathologists as negative (0), weak (1), moderate (2), and strong (3).

### Statistical analyses

The statistical analysis was performed using GraphPad Prism 8. Three independent runs of each experiment were conducted to measure the expression differences between the two groups. The Student’s t-test was applied to compare these differences, and Spearman’s rank correlation coefficient was utilized to investigate the correlation between rank variables. Statistical significance was determined by a p-value of less than 0.05.

## Results

### VISTA mRNA expression according to clinicopathological parameters of breast cancer

**Figure 1:**
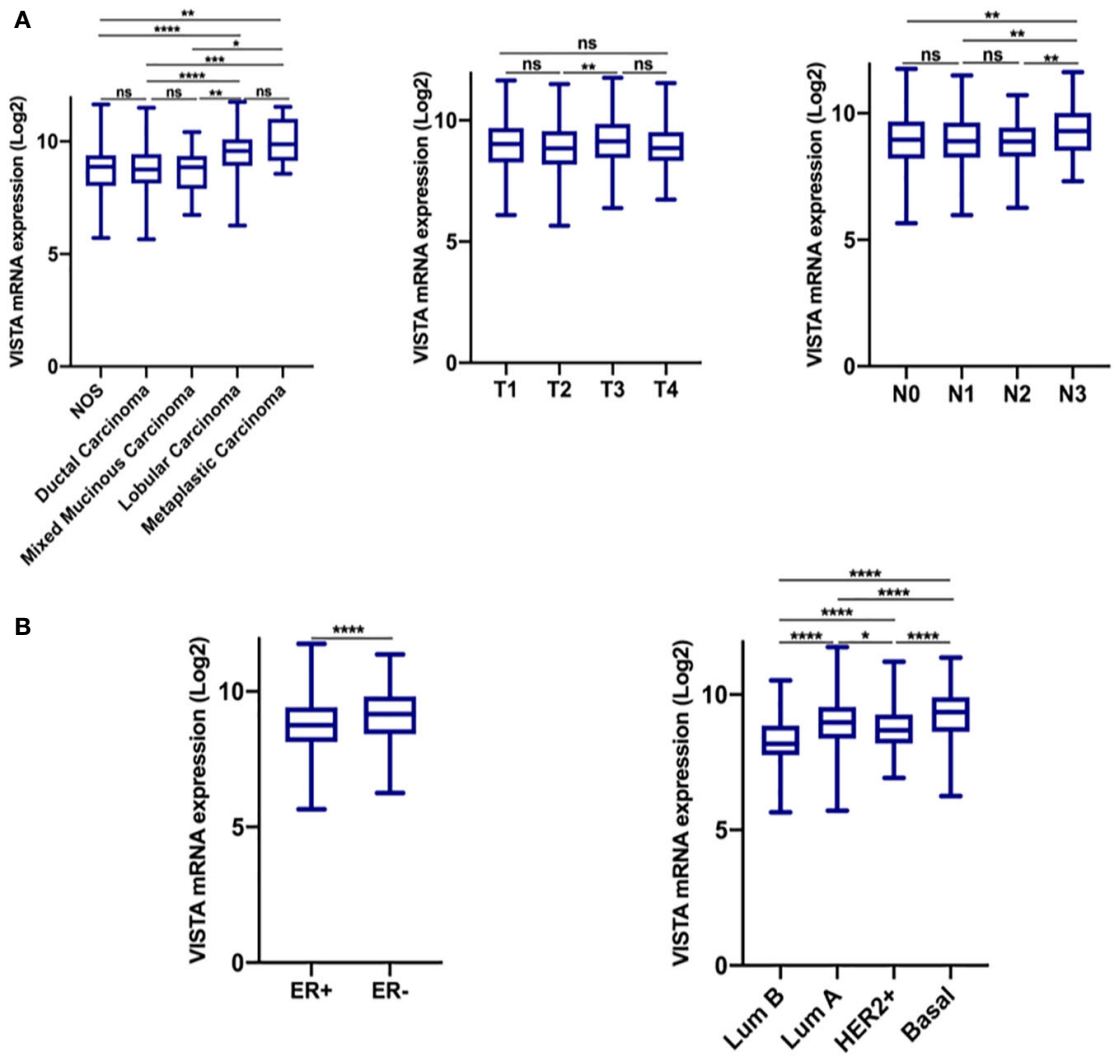
VISTA expression is significantly up-regulated in the breast cancer microenvironment. **(A)** VISTA expression in the TCGA dataset according to histological type, tumor size, and lymph node status. **(B)** VISTA expression according to ER status, and molecular subtype. *Indicates p-value < 0.05, **Indicates p-value < 0.01, ***Indicates p-value < 0.001; ****Indicates p-value < 0.0001. ns indicates that the test is non-significant..

**Figure 2:**
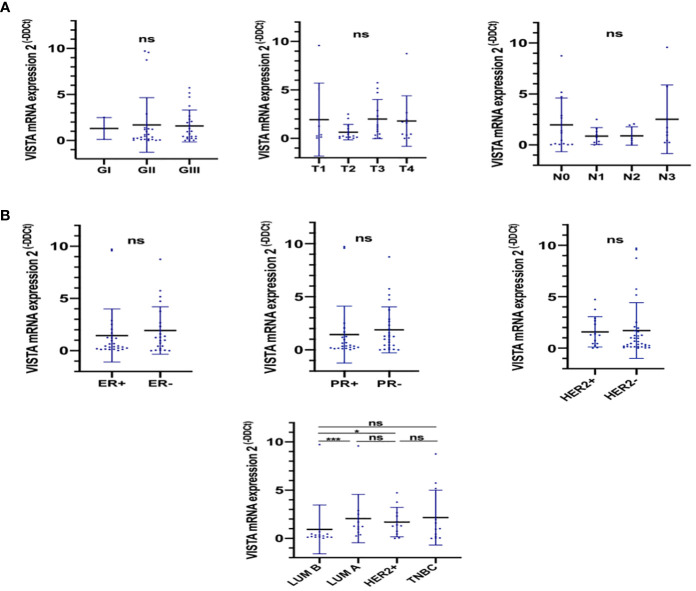
VISTA expression according to different parameters of breast cancer. RT-PCR assays were performed to evaluate VISTA expression. **(A)** VISTA expression according to the tumor grade, tumor size, and lymph node status. **(B)** VISTA expression according to ER, PR, HER2 status and breast cancer molecular subtypes. *Indicates p-value < 0.05, ***Indicates p-value < 0.001. ns Indicates test non significant.

To evaluate VISTA gene expression and its association with clinicopathological features in breast cancer patients, we assessed VISTA transcripts in two different cohorts: breast cancer patients from the TCGA dataset (**Table 1**; **Figure 1**) and from a Moroccan cohort, including both tumor and adjacent uninvaded tissues, where real-time qRT-PCR was performed (**Figure 2**).

**Table 1:** VISTA expression according to different clinicopathological parameters on TCGA cohort.

Parameters	Number	P-value
**AGE**	<50 years (293) >= 50 years (789)	0,3904
**HISTOLOGICAL TYPE**	NOS (75) Ductal Carcinoma (780) Lobular Carcinoma (201) Mixed Mucinous Carcinoma (17) Metaplastic Carcinoma (8)	**<0,0001**
**STAGE**	SI (180) SII (615) SIII (249) SIV (19)	0,5935
**MOLECULAR SUBTYPE**	Luminal B (197) Luminal A (499) HER2+ (78) BASAL (171)	**<0,0001**
**TUMOR STAGE**	T1 (276) T2 (627) T3 (137) T4 (39)	**0,0074**
**LYMPH NODE**	N0 (512) N1 (355) N2 (119) N3 (76)	**0,0176**

VISTA, V-domain Ig suppressor of T-cell activation; HER2, human epidermal growth factor receptor 2(-enriched). The values in bold signify the significance of the p-value (statistical).

Based on findings from the TCGA cohort (**Figure 1A**), elevated VISTA expression was associated with lobular and metaplastic histological subtypes, larger tumors size (T3 vs. T2; p= 0,0018), significant node invasion (N3 vs. N0, N1, and N2 status; p= 0.01), and negative ER status (p < 0.0001). However, in qRT-PCR results, the VISTA transcript was significantly lower in Luminal B compared to Luminal A and HER2^+^ molecular subtypes (**Figure 2B**). Collectively, these results demonstrated an association of VISTA expression with aggressive clinicopathological features of breast cancer at the transcriptomic scale.

### VISTA protein exhibited high expression levels in breast tumor tissues compared to adjacent uninvaded tissues

To support our findings on VISTA gene expression, we conducted an immunohistochemistry analysis of VISTA protein using 51 samples from the same patients. Additionally, we examined the expression of this protein in tumor tissues versus uninvaded adjacent tissues of 8 breast cancer patients for comparison.

**Figure 3:**
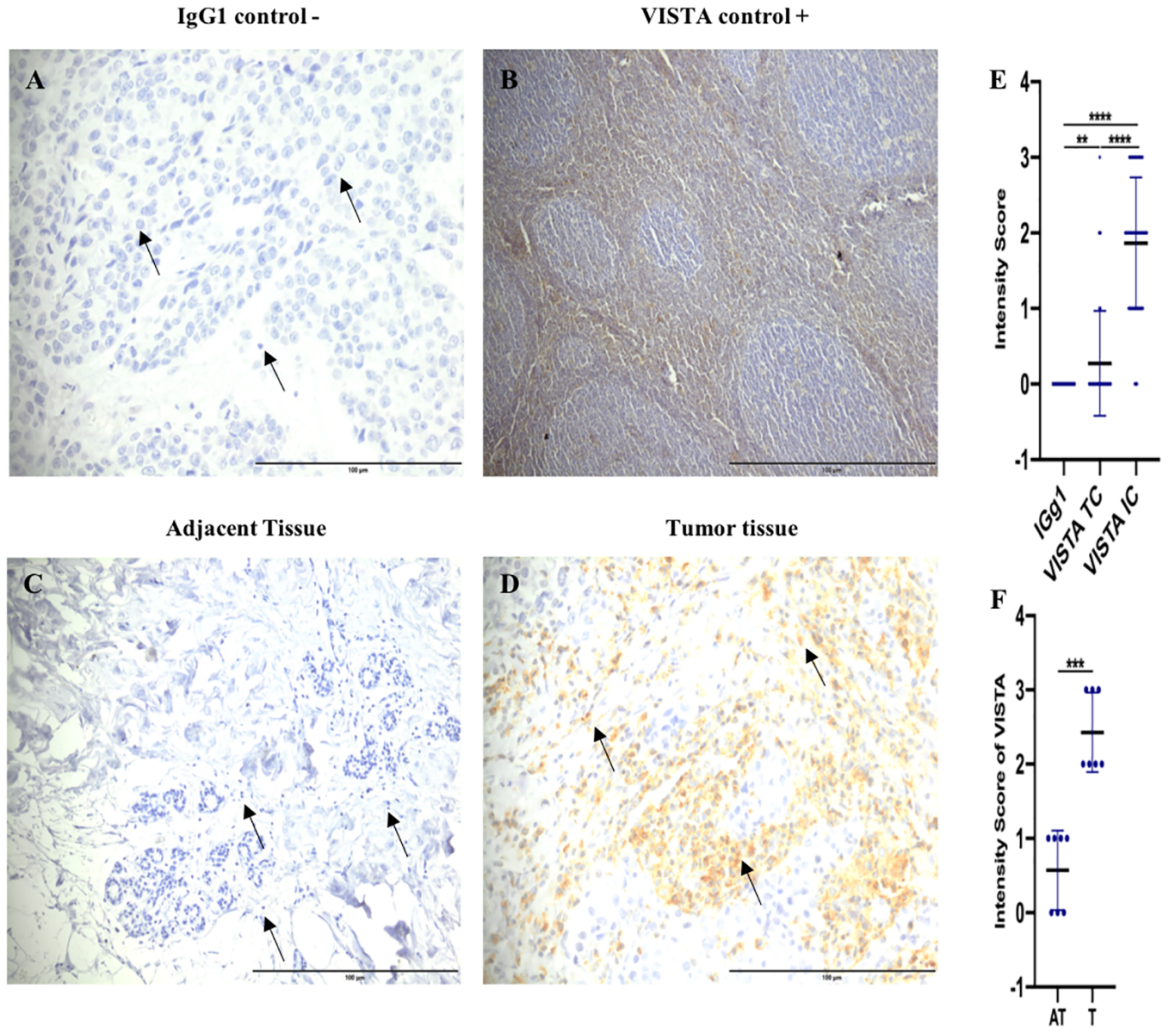
Elevated VISTA expression in immune cells of tumor tissues compared to adjacent uninvaded tissue. **(A)** Negative control staining using Mouse IgG1 Isotype Control (magnification ×40) in breast cancer patient. **(B)** Tonsil tissue as a positive control (Original magnification, × 10). **(C)** VISTA negative staining on adjacent tissues (magnification ×20). **(D)** VISTA-positive staining on tumor tissues (magnification ×40) **(E)** VISTA expression levels in immune cells, and tumor cells in breast cancer patients. **(F)** VISTA immunostaining intensity in the tumor microenvironment (Tumor (T) and Adjacent tissue (AT); (n=8)) by IHC. Scale bare 100 µm. **Indicates p-value < 0.01, ***Indicates p-value < 0.001, ****Indicates p-value < 0.0001.

An IgG1 antibody was used as a negative isotype control (**Figure 3A**). We also utilized Tonsil as a positive control tissue, which showed positive labeling of VISTA protein (**Figure 3B**). Our findings indicated that VISTA exhibited cytoplasmic and/or to membrane staining in all samples (**Figures 3C, D**). Strikingly, VISTA staining was significantly upregulated on immune cells (94%) compared to tumor cells (p <0.0001) (**Figure 3E**). Furthermore, our results indicated that VISTA was highly expressed in tumor tissues compared to uninvaded adjacent tissues (p= 0,0006) (**Figure 3F**). Taken together, these findings demonstrate overexpression of VISTA protein on immune cells in breast tumor tissues.

### VISTA protein is associated with aggressive traits of breast cancer.

**Figure 4:**
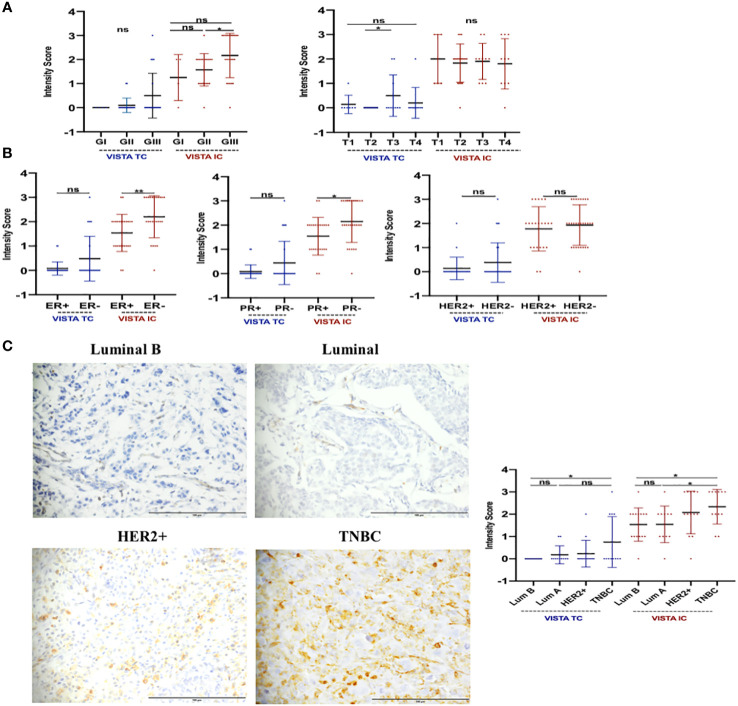
VISTA protein could be implicated in tumor progression of breast carcinoma. IHC assay was used to evaluate this expression. **(A)** Tumor grade and size might be regulated by VISTA expression. **(B)** VISTA upregulation in patients with ER, PR status, and breast cancer aggressive molecular subtypes (HER2+ and TNBC). **(C)** VISTA staining by Luminal B and A (magnification ×40), HER2+ (magnification ×40) and TNBC (magnification ×40) molecular subtypes. Scale bare 100 µm. *Indicates p-value < 0.05, **Indicates p-value < 0.01. ns Indicates test non-significant.

To validate the transcriptomic findings at the proteomic level, we examined the expression of the VISTA protein in clinicopathological parameters of 51 breast cancer patients using immunohistochemistry (as shown in **Table 2**). As illustrated in **Figure 4**, the expression of VISTA on immune cells was found to be positively associated with tumor grade (Grade III vs. Grade II, p=0.0161), as well as with ER (p= 0.0048) and PR (p= 0.0116) negative status. On the other hand, the expression of VISTA on tumor cells was significantly elevated in patients with larger tumor sizes (T3 vs. T2, p=0,0366). In addition, TNBC showed a significant upregulation of VISTA protein on both immune and tumor cells (p-value < 0.05) (**Figure 4**).

**Table 2:** VISTA expression on immune cells in breast cancer patients and their clinical characteristics.

Parameters	Number	P-value
**AGE**	<50 years (23) >=50 years (26)	0,4269
**HISTOLOGICAL TYPE**	NOS (37) Lobular Carcinoma (3) Mucinous Carcinoma (3) Micropapillary carcinoma (3) Metaplastic Carcinoma (3)	**0,0092**
**GRADE**	GI (4) GII (21) GIII (24)	**0,0245**
**TUMOR STAGE**	Luminal B (15) Luminal A (11) HER2+ (13) TNBC (12)	**0,0433**
**TUMOR STAGE**	T1 (7) T2(18) T3(10) T4 (10)	0,9661
**LYMPH NODE**	N0 (6) N1(10) N2(14) N3(6)	0,1205
**ER STATUS** **PR STATUS**	ER- (25), ER+ (26) PR- (27), PR+ (24)	**0,0048** **0,0116**

VISTA, V-domain Ig suppressor of T-cell activation; HER2, human epidermal growth factor receptor 2(-enriched), TNBC, Triple negative breast cancer; ER, estrogen receptor; PR, progesterone receptor. The values in bold signify the significance of the p-value (statistical).

Our observations on both transcriptomic and proteomic levels suggest that VISTA expression is associated with the most aggressive characteristics of breast cancer.

### VISTA expression correlates with epithelial-mesenchymal transition (EMT) markers in breast cancer

**Figure 5:**
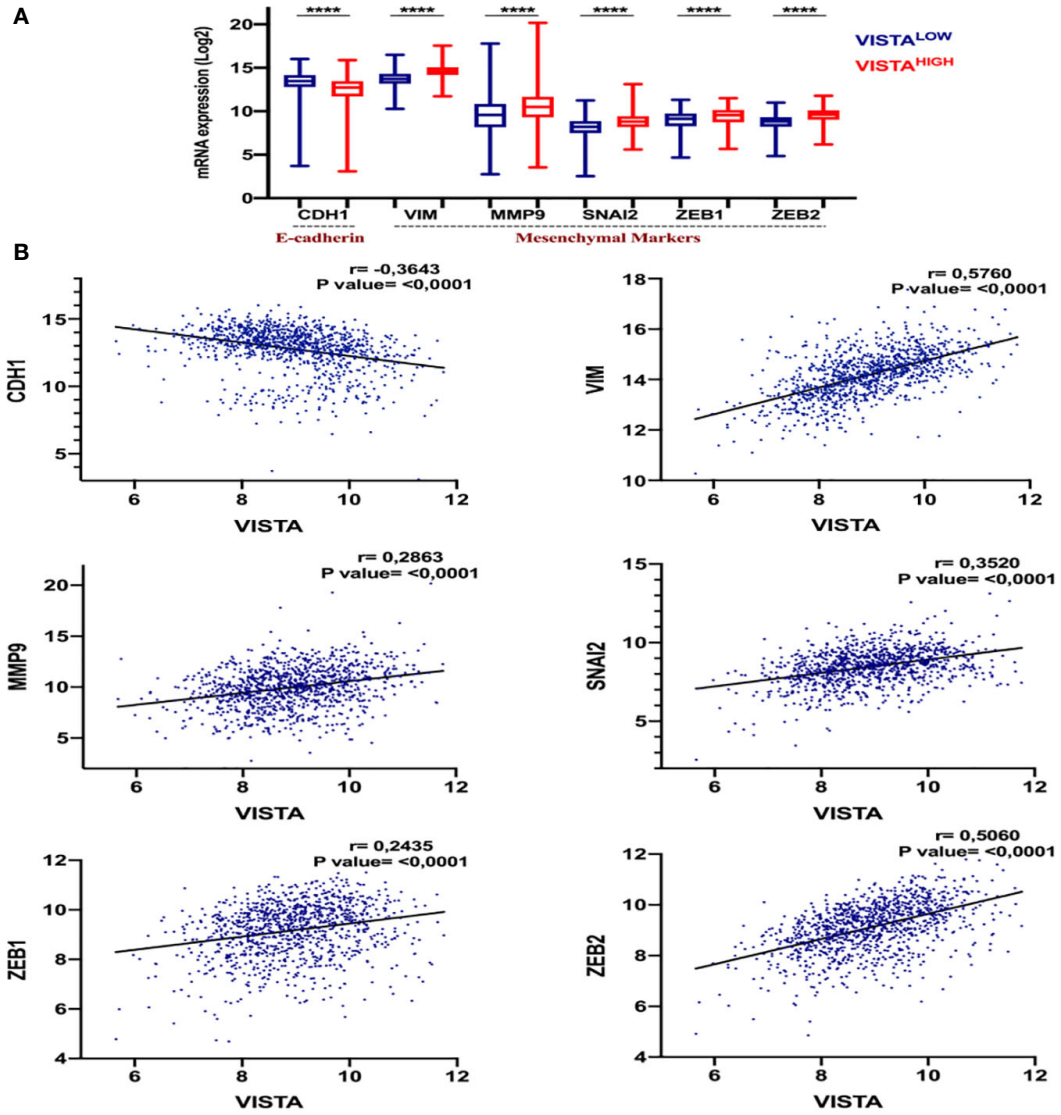
The potential association of VISTA expression and the Epithelial-mesenchymal transition (EMT) in breast cancer patients. **(A)** The expression levels of EMT genes in High versus Low VISTA groups in the TCGA cohort. The p-value is 0.05. **(B)** The correlation of VISTA expression with the EMT markers. ****Indicates p-value < 0.0001.

To evaluate the impact of VISTA on tumor progression and metastasis, we analyzed its correlation with the EMT markers in breast cancer patients. In the TCGA dataset, we assessed the expression of key epithelial marker E-cadherin (CDH1) and mesenchymal markers, including vimentin (VIM), MMP9, SNAI2, ZEB1, and ZEB2, about VISTA expression in breast cancer patients using Spearman correlation analysis (**Figure 5**). We found that VISTA expression correlated negatively with CDH1 (r= -0,3114, p <0.001) and positively with all the mesenchymal markers in breast cancer patients (**Figure 5B**). On note, EMT signature analysis according to molecular subtype, stages and tumor size stratification presented similar results patterns (**Figure Supplementary data S1**, **S2**, **S3**).

These results suggest that VISTA may contribute to breast cancer progression and metastasis, particularly in aggressive subtypes.

### VISTA and PD1 expression present a positive correlation in breast cancer patients.

**Figure 6:**
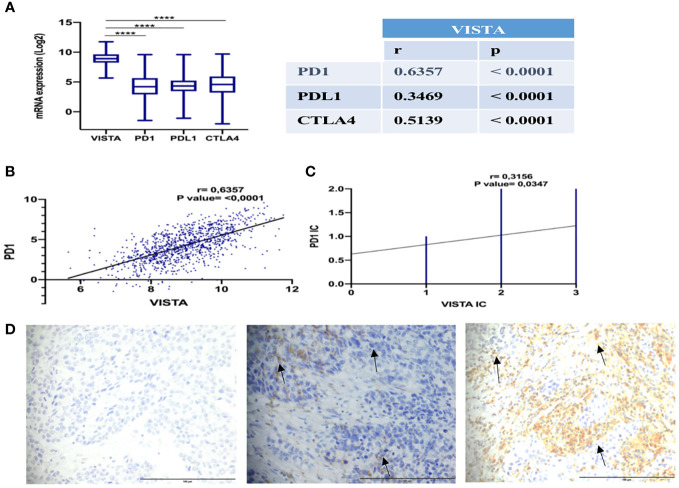
Positive correlation of VISTA and PD-1 expression in breast cancer patients. **(A)** Expression and correlation of VISTA mRNA with PD-1, PDL-1, and CTLA-4 in TCGA dataset. **(B)** Correlation of VISTA and PD-1 in TCGA cohort (r=0,4605, P-value <0,001). **(C)** Correlation of VISTA and PD-1 in immune cells by IHC in the Moroccan cohort (r=0,3156, P-value= 0.0347). **(D)** IgG1 negative control isotype staining in a TNBC patient is represented on the left, followed by PD-1 positive staining in the middle, and VISTA positivity staining on the right (magnification ×40). Scale bare 100 µm. ****Indicates p-value < 0.0001. The black arrows signify VISTA and PD1 expression on immune cells.

**Figure 7:**
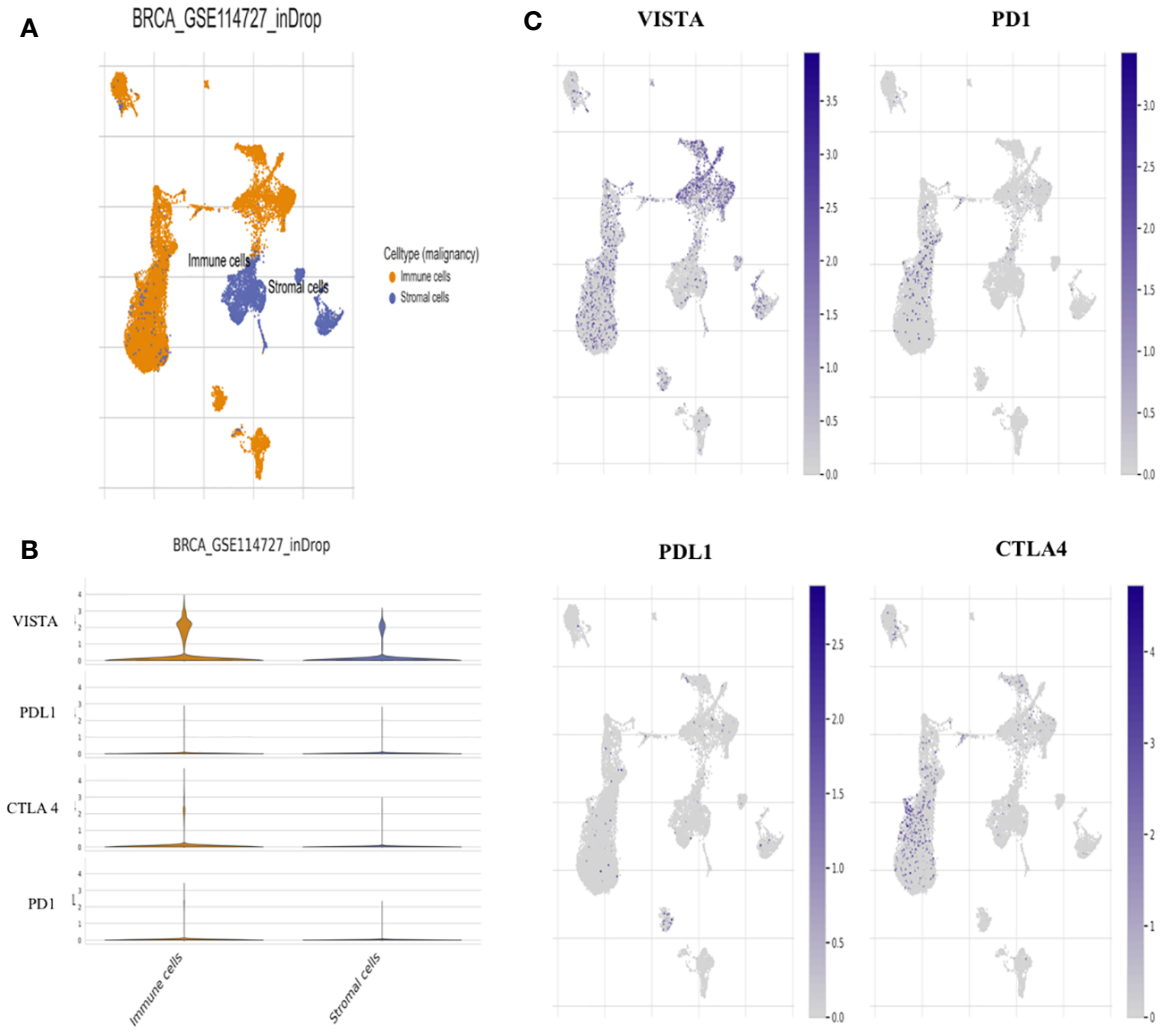
Single-cell analysis for VISTA based on Tumor Immune Single-cell Hub (TISCH) database. **(A)** Uniform manifold approximation and projection (UMAP) plots showing the Breast cancer cell landscape based on GSE114727_inDrop. **(B)** Violin plots for the expression of VISTA, PDL1, CTLA4, and PD1 on Immune and stromal cells based on GSE114727_inDrop. **(C)** UMAP plots illustrating the expression of VISTA, PD1, PDL1, and CTLA4 clusters based on GSE114727_inDrop.

To assess the impact of VISTA expression on the establishment of an immunosuppressive microenvironment in breast cancer, a comparison was made with the main inhibitory immune checkpoints used in immunotherapy, namely PD-1, PDL-1 and CTLA-4. Using the TCGA dataset, the analysis revealed significant overexpression of VISTA in mammary tumor (**Figure 6A**). Analysis of the Tumor Immune Single Cell Hub (TISCH) database (**Figure 7**) supported these findings by demonstrating that VISTA was highly expressed on immune cells relative to stromal cells and that its expression was significantly more favorable than that of PD-1, PDL-1, and CTLA-4 in breast cancer. Subsequently, we examined the correlation between VISTA expression and its homologous molecule PD-1 using results from two independent cohorts, namely the Moroccan cohort (by IHC) and the TCGA dataset.

In both cohorts, VISTA and PD1 showed a positive correlation (**Figures 6B–D**), suggesting that a treatment targeting both immune checkpoints could potentially improve the response rate to immunotherapy in breast cancer patients.

## Discussion

In Breast cancer, approved immunotherapy is restricted to metastatic PDL1+ TNBC tumors, with only 13% of patients receiving anti-PDL1 treatment (Atezolizumab) experiencing a significant response rates, longer overall survival, and higher immune cell infiltration (46–48). Therefore, the failure of PD1/PDL1 immunotherapy necessitates the consideration of alternative molecules as drug candidates for breast cancer treatment. Based on these clinical observations, VISTA expression and immunosuppressive effects in breast cancer patients were investigated using a Moroccan cohort and TCGA dataset, which included all molecular subtypes.

Of note, our first result revealed that VISTA is overexpressed in tumor tissues compared to adjacent uninvaded tissues, particularly in immune cells (94%). Based on other findings, invasive ductal carcinoma (IDC) of breast cancer exhibits overexpression of VISTA compared to adjacent normal tissues, with an expression level of 29.1% in immune cells and 8.2% in tumor cells (42, 43). Similar results have been observed in various malignancies notably ovarian, endometrial, and pancreatic cancers, where VISTA was highly expressed on tumor cells compared to adjacent cells (32, 49). In contrast, VISTA expression was detected in 29.5% of the hepatocellular carcinoma (HCC) samples, representing 16.4% of the staining on tumor cells and 16.9% on immune cells (50).

Overall, these results suggest that VISTA may be a promising target for breast cancer immunotherapy, particularly in IDC. However, further studies are needed to explore the potential of VISTA-targeted therapies in breast cancer treatment.

Additionally, our results demonstrated that VISTA expression is positively associated with aggressive subtypes, including lobular and metaplastic carcinoma. In breast cancer patients, a high level of VISTA is typically associated with high grade, large tumor size, lymph node involvement, ER and PR negative status, and TNBC molecular subtype. Previous research has shown that VISTA expression in breast cancer is linked to ER and PR negative status, as well as HER2+ and basal molecular subtypes (43). Furthermore, it has been reported that VISTA expression positively correlates with lymph node invasion in oral squamous cell carcinoma (51). However, Xie and colleagues’ study found that VISTA expression did not have a significantly correlation with tumor size or lymph node status in patients with gastric and colorectal cancer (36, 52).

These results suggest that VISTA expression is associated with the most aggressive clinical features and may contribute to tumor progression. Therefore, VISTA blockade could be a promising therapeutic option for breast cancer patients.

Multiple studies have demonstrated that targeting multiple immune checkpoints in malignant diseases leads to the functional synergy that activates the anti-tumor immune response, considerably increasing patient response rates compared to monotherapy (53, 54). Liu and colleagues discovered that VISTA and PD-1 exert nonredundant immune regulatory functions and synergistically regulate T-cell responses in the KO mice model (44). Our findings revealed that VISTA is highly expressed in the breast tumor microenvironment and is more correlated with PD-1 than PDL-1 or CTLA-4.

Thus, combined therapy involving VISTA and PD-1 blockade in breast cancer patients could improve treatment outcomes.

Growing evidence supports the role of EMT in tumor cell progression, invasion, and metastasis (11, 55, 56). These phenotypic shifts are driven by alterations in gene expression mediated by mesenchymal markers SNAIL, TWIST, and ZEB1, leading to increased levels of vimentin and decreased levels of epithelial markers E-cadherin (57, 58). Additionally, EMT contributes to cancer recurrence and drug resistance by acquiring stem cell-like properties (59). Similar studies have shown that EMT increases the expression of immune checkpoint inhibitors in numerous cancer, which minimizes immune cell infiltration into the tumor microenvironment, leading to increased immunosuppression (60, 61). The EMT phenotype is positively correlated with PD-L1, PD-L2, PD-1, TIM-3, B7-H3, BTLA, and CTLA-4 expression, as well as CD4^+^ Foxp3^+^ Tregs expansion in non-small cell lung cancer (NSCLC) (62). Several studies have reported the impact of EMT on breast cancer prognosis, in which epithelial markers are downregulated, while mesenchymal markers and invasive-related genes are upregulated in basal-like tumor (11). Moreover, PDL-1 expression, Tregs, M2 macrophages, and exhausted CD8+ T cell infiltration were positively correlated with mesenchymal markers in breast cancer stroma (63).

As shown by our study, VISTA correlates negatively with the epithelial marker E-cadherin (CDH1) and positively with mesenchymal genes (VIM, MMP9, SNAI2, and ZEB1).

In light of our findings, VISTA correlates negatively with the epithelial marker E-cadherin (CDH1) and positively with mesenchymal genes (VIM, MMP9, SNAI2, and ZEB1). Therefore, these results suggest that VISTA expression may be associated with metastatic potential due to the EMT process in breast carcinoma.

Overall, our findings indicate that VISTA represents a promising therapeutic target in breast cancer, encouraging further clinical investigation in patients with breast cancer.

## Conclusion

Based on our study, we can conclude that VISTA may be involved in breast cancer aggressiveness, given its association with the ER, PR, HER2+, and TNBC molecular subtypes. Moreover, its expression is closely related to the expression of EMT markers. Thus, it would be necessary to develop a combination therapy targeting both molecules (VISTA and PD1) to counteract immunosuppression and stimulate an effective anti-tumor response.

## Data availability statement

The datasets presented in this study can be found in online repositories. The names of the repository/repositories and accession number(s) can be found in the article/**Supplementary Material**.

## Ethics statement

The studies involving human participants were reviewed and approved by the Ethics Committee for Biomedical Research (CERB), Casablanca, with the approval code 28/5. The patients/participants provided their written informed consent to participate in this study.

## Author contributions

IRE collected, analyzed, and interpreted data, and wrote the manuscript. BZ and SA collected, and analyzed data, and revised the manuscript. MK and IRA analyzed data. AB designed research, analyzed and interpreted data, wrote and revised the manuscript, and supervised the study. All authors contributed to the article and approved the submitted version.
