# Supplementary figures and images for: High VISTA expression is linked to a potent epithelial-mesenchymal transition and is positively correlated with PD1 in breast cancer
Source: Front Oncol. 2023 Apr 20;13:1154631. doi: 10.3389/fonc.2023.1154631 (PMC10157209; doi:10.3389/fonc.2023.1154631)

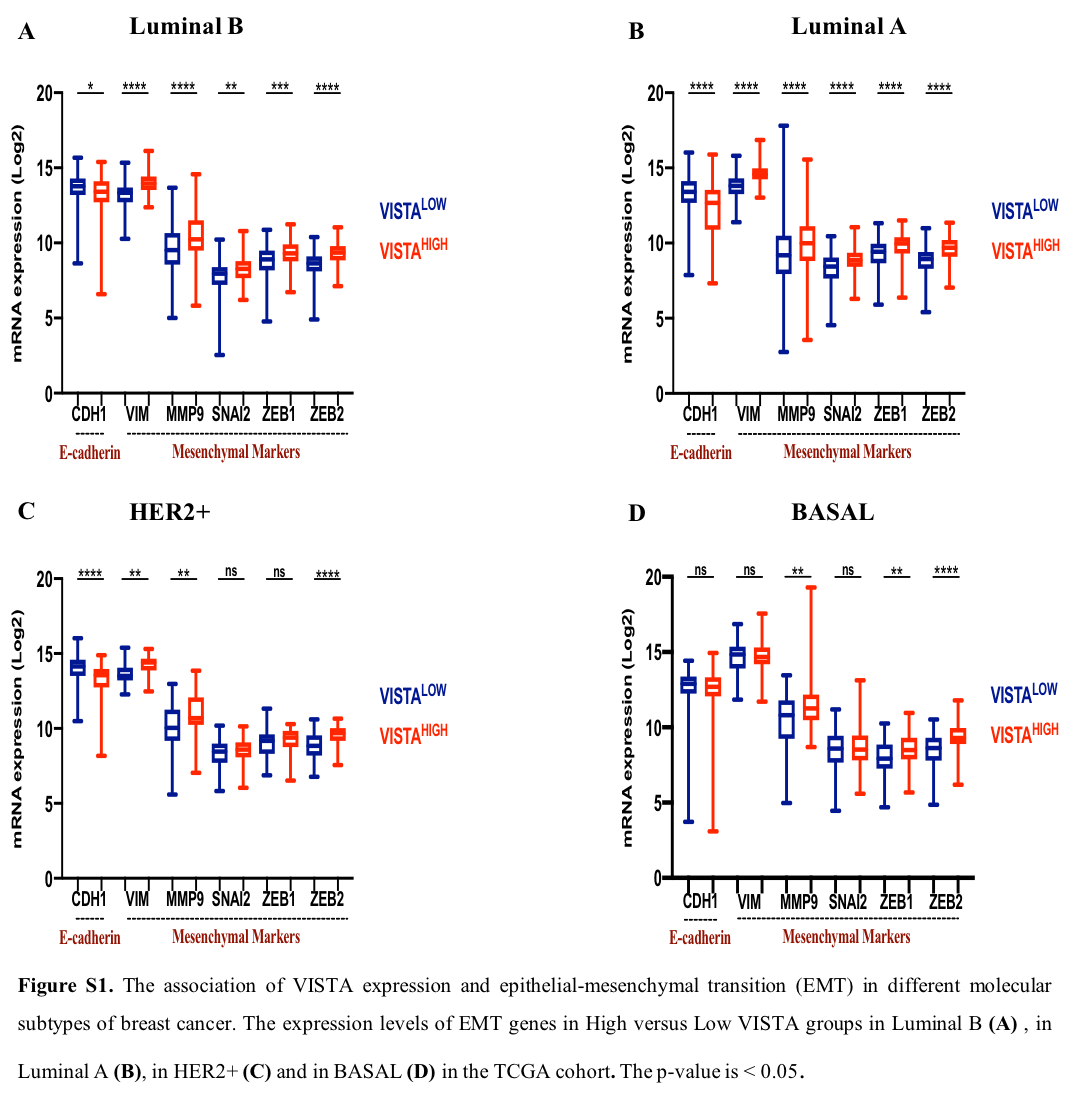

Supplement: Supplementary file 1 [file Image_1.tiff]

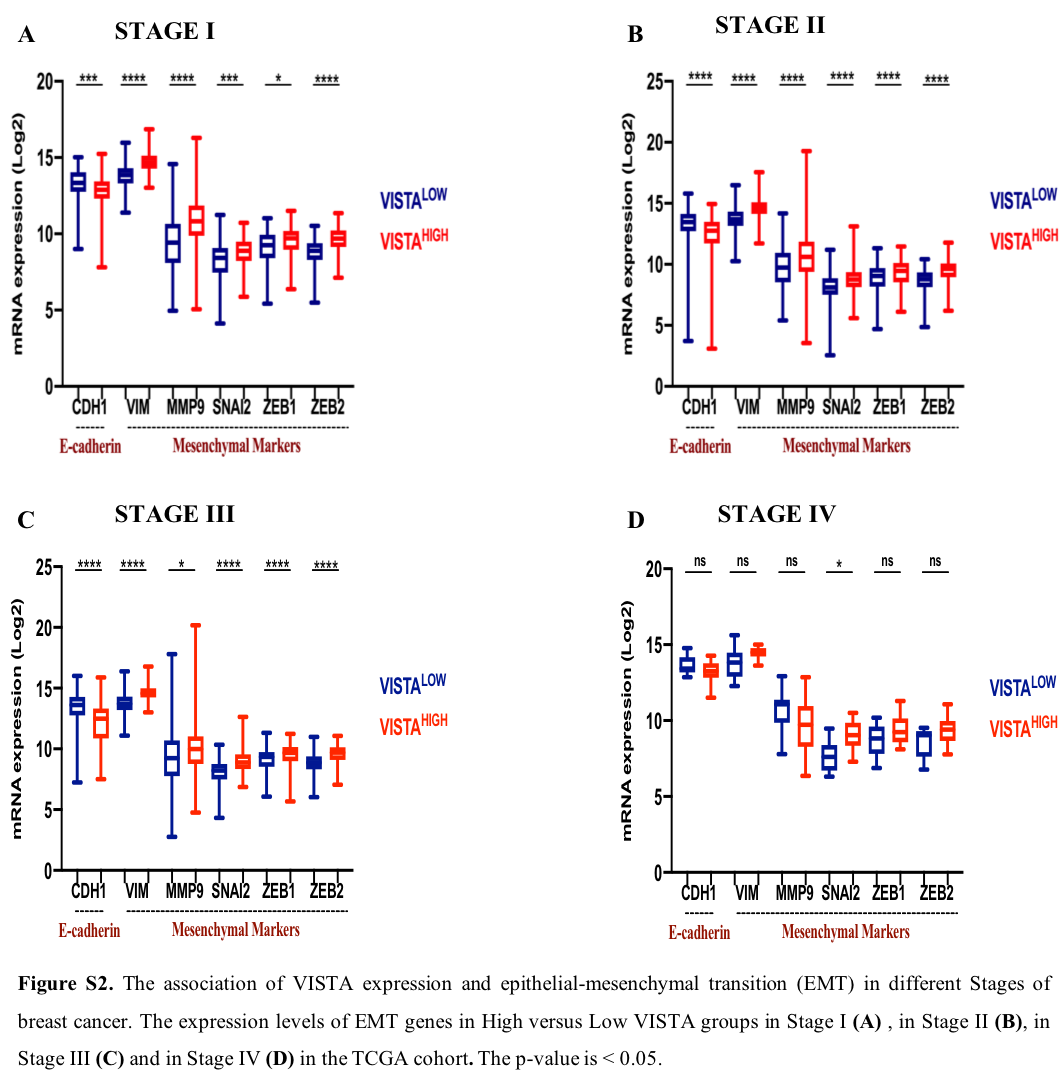

Supplement: Supplementary file 2 [file Image_2.tiff]

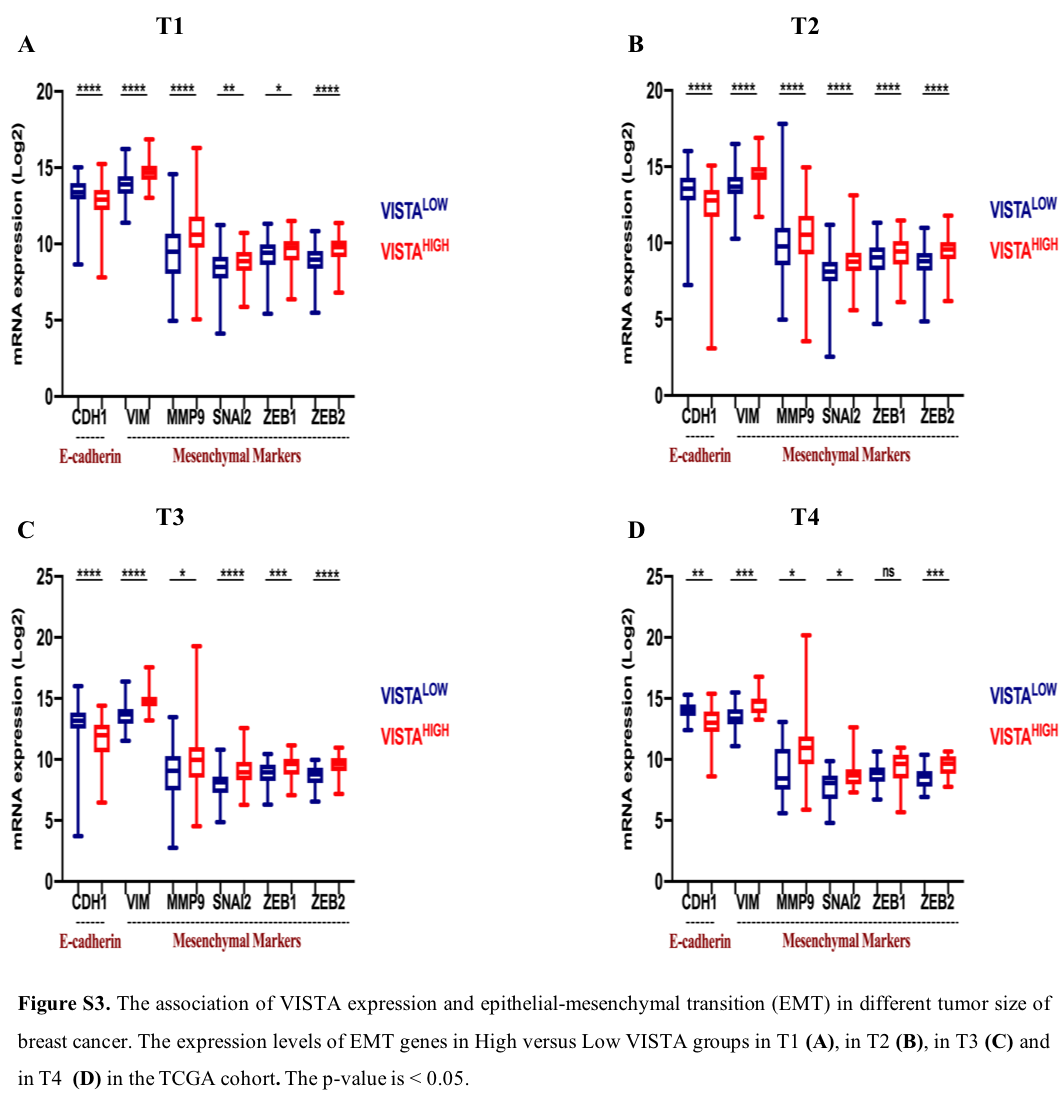

Supplement: Supplementary file 3 [file Image_3.tiff]
